# Stabilization of Quinapril by Incorporating Hydrogen Bonding Interactions

**DOI:** 10.4103/0250-474X.57288

**Published:** 2009

**Authors:** B. N. Roy, G. P. Singh, H. M. Godbole, S. P. Nehate

**Affiliations:** Lupin Ltd. (Research Park), 46A, 47A - Nande Village, Mulshi Taluka, Pune-411 042, India

**Keywords:** Quinapril, solid state, crystal structure, stabilization, stability, solvates, co-crystal

## Abstract

In the present study stability of various known solvates of quinapril hydrochloride has been compared with nitromethane solvate. Nitromethane solvate was found to be more stable compared to other known solvates. Single crystal X-ray diffraction analysis of quinapril nitromethane solvate shows intermolecular hydrogen bonding between quinapril molecule and nitromethane. Stabilization of quinapril by forming strong hydrogen bonding network as in case of co-crystals was further studied by forming co-crystal with tris(hydroxymethyl)amino methane. Quinapril free base forms a stable salt with tris(hydroxymethyl)amino methane not reported earlier. Quinapril tris(hydroxymethyl)amino methane salt found to be stable even at 80° for 72 h i.e. hardly any formation of diketopiperazine and diacid impurity. As expected single crystal X-ray diffraction analysis reveals tris(hydroxymethyl)amino methane salt of quinapril shows complex hydrogen bonding network between the two entities along with ionic bond. The properties of this stable salt - stable in solid as well as solution phase, might lead to an alternate highly stable formulation.

Cyclization of dipeptides to diketopiperazine is well documented in the literature[1–4]. Angiotensin-converting enzyme (ACE) inhibitors, a class of drug molecules effective in treatment of cardiovascular dysfunctions, are also dipeptides, and hence are prone to cyclization easily to diketopiperazine. Formation of diketopiperazine is a major stability issue of concern to the potent ACE inhibitors such as enalapril, moexipril, lisinopil, perindopril, ramipril and quinapril.

Quinapril hydrochloride[5–7] (‘I’ in fig. 1) easily forms the corresponding diketopiperazine on heating and, in solution, rapidly cyclize to diketopiperazine impurity (DKP impurity, ‘II’ in fig. 1), compared to the other ACE inhibitors during storage and processing. The diketopiperazine impurity is formed either during the manufacture of quinapril hydrochloride, or during drying/further formulation of quinapril hydrochloride. The said impurity once formed is difficult to remove by conventional separation techniques including fractional crystallization.

**Fig. 1:**
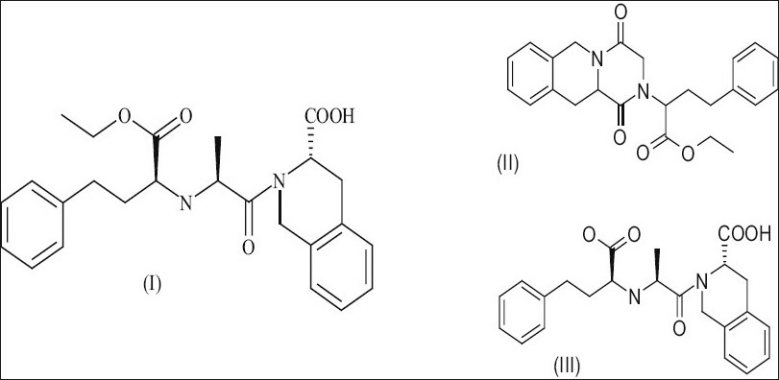
Quinapril and its impurities. I: Quinapril, II: Diketopiperazine impurity of quinapril, III: Diacid impurity of Quinapril.

In case of various ACE inhibitors like enalapril, moexipril and perindopril the tendency of cyclization to the corresponding diketopiperazine impurity has been minimized by forming the acid or base addition salts. The techniques employed to stabilize other ACE inhibitors were found to be unsuccessful in case of quinapril. On storage specifically at elevated temperatures quinapril or its salts have been found to cyclize to DKP and also to diacid (‘III’ in fig. 1) through hydrolysis.

The product obtained by employing the process described in US Patent No. 4,344,949 for quinapril hydrochloride results in an amorphous solid. Guo *et al.*[8] describe stability and chemical degradation of amorphous quinapril hydrochloride. Due to the amorphous nature it gets readily converted to the diketopiperazine impurity at 80°. Studies on crystalline quinapril hydrochloride acetonitrile solvate shows that during heating of samples, initial evaporation of solvent at 60° occurs which is followed by cyclization to the diketopiperazine impurity by loss of water and hydrogen chloride.

Desolvation of stoicheometric solvates most of the times lead to a different crystal structure or results in a disordered or amorphous state[9], with some exceptions[10–13]. In case of quinapril hydrochloride solvates, the product gets totally converted to an amorphous solid on desolvation. On heating, solid quinapril hydrochloride gets converted to the diketopiperazine impurity, in the solid stage escape of hydrogen chloride is the rate limiting step, and in case of solution phase, formation of quinapril zwitterions is the rate limiting step, which can be accelerated by increasing the pH of the solution[14]. The schematic representation of various steps involved in formation of diketopiperazine impurity as described by Li *et al*[14] is provided in fig. 2.

**Fig. 2:**
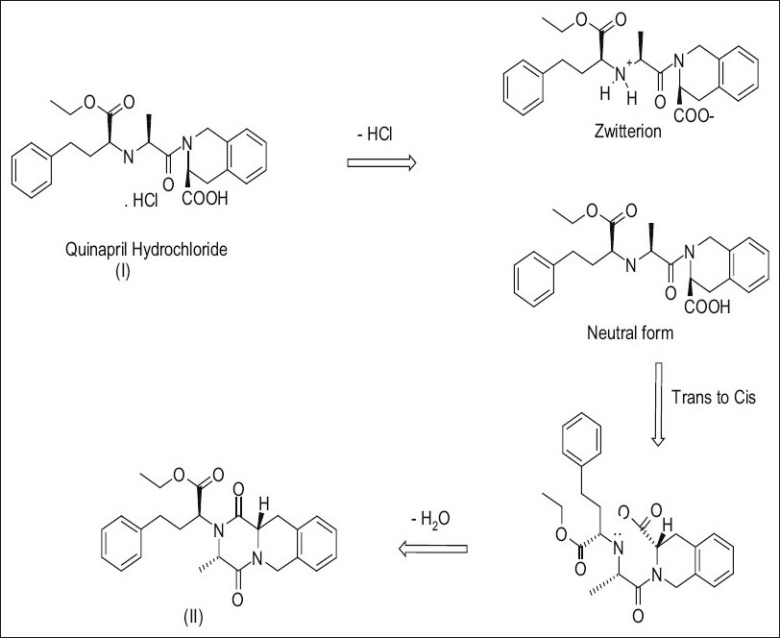
Steps in the degradation of quinapril hydrochloride. Schematic representation showing various steps involved during degradation of quinapril hydrochloride. - HCl: escape of hydrogenchloride, - H_2_O: escape of water molecule

Some approaches to stabilize quinapril and/or quinapril hydrochloride are provided by Harris *et al*[15], Sherman[16], Klokkers *et al*[17,18], Li *et al*[14,19] and Goel *et al*[20]. Although attempts to improve the stability of solid quinapril have been partially successful, no work has been reported for improvement of solution phase stability.

Intermolecular interactions between quinapril molecule and solvent molecule(s) would minimize the intramolecular interactions, which are responsible for formation of the diketopiperazine impurity. The crystalline structure formed in case of solvates would minimize the mobility of molecules which is more in case of amorphous solids[8]. This postulation lead to our earlier work[21] related to quinapril hydrochloride nitromethane solvate. Nitromethane solvate is comparatively more stable than other known solvates *e.g.* acetonitrile solvate, due to hydrogen bonding interaction between quinapril hydrochloride molecule and nitromethane. Apparently, acetonitrile solvate of quinapril hydrochloride shows inferior stability to that of nitromethane solvate because no hydrogen bonding has been observed in single crystal X-ray diffraction pattern in the former case.

In view of the above, attempts have been made to design a system wherein addition of a molecule could improve hydrogen bonding which in turn could impart stability to quinapril hydrochloride, preferably the free base. One such approach is to form a ‘co-crystal’ with a suitable molecule, which is generally recognized as safe to human beings (a GRAS molecule).

The term ‘co-crystal’[22–29] represents a long known class of compounds, a prototypal is quinhydrone and is broadly defined as “a mixed crystal of crystals that contains two different molecules”. Supramolecular chemistry and crystal engineering concept defines it as a consequence of a molecular recognition event between different molecular species. Basically co-crystals are made from reactants that are solids at ambient temperature; this fact distinguishes solvates/hydrates from co-crystals. In solvates, at least one component should be a liquid at ambient temperature; in hydrates, it is water[22].

## MATERIALS AND METHODS

All solvents used for experiments were of HPLC grade. Tris(hydroxymethyl)amino methane and *tert*-butylamine were purchased from M/S S. D. Fine Chemicals Ltd., Mumbai, India. ^1^H NMR spectra were recorded on a Bruker 400MHz spectrometer, powder X-ray diffraction was recorded on PANalytical B.V., Netherlands model PN3040/60X'Part Pro., FT IR spectra were recorded on a Perkin-Elmer model spectrum 100, thermal analyses were done on a Mettler Toledo DSC 821e.

The solvates of quinapril hydrochloride with acetonitrile[20], acetone[30], toluene[31] and methyl formate[30] were prepared by the process reported in literature. The method as published by Singh *et al*[21] was utilized to prepare the nitromethane solvate. Quinapril magnesium salt and quinapril hydrochloride were prepared according to the process reported by Sharman[16] and Hoefle[5] respectively.

### Preparation of nitromethane solvate of (S,S,S)2-{2-[(1-ethoxycarbonyl-3-phenylpropyl) amino]-1-oxopropyl]-1,2,3,4-tetrahydroisoquinoline-3-carboxylic acid hydrochloride:

Preparation of (S,S,S)2-{2-[(1-ethoxycarbonyl-3-phenylpropyl)amino]-1-oxopropyl]-1,2,3,4-tetrahydroisoquinoline-3-carboxylic acid hydrochloride. (quinapril hydrochloride) was achieved by following process: (S,S,S)-2-[2-{(1-ethoxycarbonyl)-3-phenylpropyl)amino]-1-oxopropyl]-1,2,3,4-tetrahydro-3-isoquinoline carboxylic acid benzyl ester maleate salt (quinapril benzyl ester maleate salt) 25 g (0.0388 mol) was dissolved in a mixture of 125 ml water and 125 ml dichloromethane. The pH of the solution was adjusted between 7.5 and 8.5 by addition of aqueous ammonia. Reaction mixture was stirred for 30 min, the organic phase separated and washed with 50 ml water. The organic phase was evaporated under reduced pressure below 40°, the free base of quinapril benzyl ester was obtained.

The above obtained residue of quinapril benzyl ester was dissolved in 140 ml ethanol to which 1.0 g of 10% Pd/C and 6.0 g of 35% hydrochloric acid were added. The reaction mass was subjected to catalytic hydrogenation at 40-60 psi pressure and 20-30°. The reaction mass was filtered and the filtrate evaporated to give crude quinapril hydrochloride.

The residue obtained was dissolved in nitromethane (125 ml) and the solvent recovered below 35° under reduced pressure. This operation was repeated till the water content of the residue was less than 0.5%. Nitromethane (125 ml) was added to the above residue of quinapril hydrochloride and the mixture was stirred at 20-25° for 10-15 min to get a clear solution. The mixture was stirred at the same temperature for 30 min and seeded with pure quinapril hydrochloride. The mass was cooled to 5-10° and stirred at the same temperature for 2 h. The reaction mass was then filtered and the wet cake was washed with nitromethane (50 ml), to give crystalline quinapril hydrochloride nitromethane solvate.

FTIR (KBr, v, cm^−1^): 3030, 2933, 2856, 2796, 1743, 1701, 1645, 1550, 1490, 1450, 1263, 1197, 1091, 756, 729, 707. Thermo gravimetric analysis showed weight loss of 10.44% due to loss of solvent between the temperature ranges of 40° to 125°, a further loss of 10.24% was observed between the range of 125 to 200° due to loss of hydrogen chloride and water leading to degradation. DSC (50.0-300.0°; 05.00°/min): integral (1): 233.25 mJ; integral (2): 245.18 mJ, onset(1): 96.99°; onset(2): 151.25°, peak(1): 107.39°; peak(2): 163.53°, endset(1): 111. 55°; endset(2): 172.43°. The specific optical rotation for quinapril hydrochloride nitromethane solvate: 9.53 degrees (C= 1 in water at 20° for sodium line). X-ray powder diffraction pattern showed peaks at about 7.28, 9.06, 11.03, 11.68, 12.23, 13.67, 13.77, 14.55, 15.99, 16.62, 16.72, 17.00, 18.14, 18.55, 18.81, 19.86, 20.03, 21.55, 21.68, 21.88, 22.13, 23.43, 23.63, 24.61, 25.28, 25.95, 26.42, 27.15, 27.35, 27.69, 28.19, 28.81, 29.31, 29.96, 30.24, 30.49, 30.64, 31.69, 32.06, 33.46, 34.06, 34.35, 34.89, 36.29, 37.42, 38.13, 39.08, 39.68±0.2 °2θ.

### Preparation of (S,S,S)2-{2-[(1-ethoxycarbonyl-3-phenylpropyl)amino]-1-oxopropyl]-1,2,3,4-tetrahydroisoquinoline-3-carboxylic acid tris(hydroxymethyl)amino methane salt:

Quinapril benzyl ester maleate salt 25 g (0.0388 mol) was converted to quinapril benzyl ester as per the procedure provided above. The residue of quinapril benzyl ester was dissolved in 250 ml ethanol. Palladium on charcoal (10%), 2.5 g, tris(hydroxymethyl)amino methane 4.7 g (0.0388 mol) and 60 ml water were added to the ethanol solution. The reaction mass was subjected to catalytic hydrogenation at 40-60 psi pressure at 20-30° for 2 h. After completion of reaction, the reaction mass was filtered and the filtrate was evaporated to dryness. To the residue, 100 ml acetonitrile was added and stirred for 30 min. The solid was filtered and dried at 40-45° for 10 h to give 22 g quinapril tris(hydroxymethyl)amino methane salt.

FTIR Spectra (KBr, cm^−1^): Please refer to Table 1. ^1^H NMR (DMSO-d_6_ + CDCl_3_): δ 7.23 (m, 9H), 5.12 (m, 1H), 4.86-4.60 (m, 3H), 4.07 (m, 2H), 3.65 (m, 1H), 3.34 (s, 6H), 3.15 (m, 2H), 2.48-2.87 (m, 2H), 1.83 (m, 2H), 1.02-1.2 (m, 6H). Thermo gravimetric analysis shows no weight loss. DSC (50.0-300.0°; 05.00 °/min): integral: -800.04 mJ/g; onset: 155.13°; peak: 156.44°, endset: 158.53°. The specific optical rotation for quinapril tris is: -24.34° (C = 1 in water at 20° for sodium D-line). X-ray powder diffraction pattern shows peaks at about 3.80, 7.57, 8.60, 9.33, 9.46, 11.35, 14.11, 14.38, 14.78, 15.16, 16.17, 17.16, 17.59, 17.74, 18.23, 18.96, 20.14, 20.34, 20.68, 20.86, 22.49, 22.74, 23.24, 23.81, 24.56, 25.24, 26.17, 26.67, 26.93, 28.53±0.2 °2θ.

**TABLE 1:** COMPARISON OF FTIR SPECTRA OF QUINAPRIL TRIS, ERBUMINE SALT AND HYDROCHLORIDE SALT

Quinapril tris salt* (cm^−1^)	Quinapril erbumine salt* (cm^−1^)	Quinapril HCl salt* (cm^−1^)
3324, 3368, 3085,	3709, 3679, 3420,	3413, 3027, 2982,
3033, 2988, 2947,	3317, 3026, 2977,	2935, 2856, 1739,
2926, 2904, 2858,	2903, 2843, 2745,	1648, 1536, 1496,
2606, 2551, 2089,	2634, 2555, 2230,	1449, 1382, 1335,
1718, 1625, 1570,	1720, 1647, 1565,	1294, 1258, 1207,
1537, 1498, 1480,	1497, 1484, 1455,	1140, 1111, 1088,
1455, 1427, 1394,	1442, 1413, 1391,	1034, 1015, 985,
1371, 1339, 1286,	1364, 1337, 1283,	935, 909, 855, 749,
1233, 1215, 1196,	1230, 1194, 1150,	701, 679, 633, 595,
1180, 1155, 1115,	1112, 1058, 1032,	492, 422
1083, 1060, 1035,	1016, 983, 942, 924,	
945, 922, 813, 743,	852, 817, 753, 744,	
654, 584, 508, 441.	713, 703, 655, 541,	
	507, 466.	

* FTIR absorption peaks observed by preparing pellet of solid dispersion of respective compound in dry potassium bromide. FTIR: Fourier Transform Infra-red Spectra; cm is centimeter; tris is tris(hydroxymethyl)amino methane; erbumine is *tert*-butylamine and HCl is hydrochloride

### Preparation of (S,S,S)2-{2-[(1-ethoxycarbonyl-3-phenylpropyl)amino]-1-oxopropyl]-1,2,3,4-tetrahydroisoquinoline-3-carboxylic acid tertiary butyl amine salt [quinapril erbumine salt]:

Quinapril benzyl ester maleate salt 25 g (0.0388 mol) was converted to quinapril benzyl ester as per the procedure provided above. The residue of quinapril benzyl ester was dissolved in 250 ml ethanol. To this solution, 2.5 g of 10% Pd/C and 4.25 g (0.0582 mol) tertiary butyl amine were added. The reaction mass was subjected to catalytic hydrogenation at 40-60 psi pressure at 20-30° for 2 h. After completion of reaction, the reaction mass was filtered at 50° and the filtrate evaporated to dryness. To the residue, 100 ml acetonitrile was added and stirred for 30 min. The solid was filtered and dried at 40-45° for 10 h. Dry weight of quinapril tertiary butyl amine salt: 20 g

FTIR Spectra (KBr, cm^−1^) were presented in Table 1. ^1^H NMR (CDCl_3_): δ 7.23 (m, 9H), 5.12 (m, 1H), 4.86-4.60 (m, 3H), 4.11 (m, 2H), 3.77 (m, 1H), 3.3 (m, 2H), 2.65 (m, 2H), 1.1.98 (m, 2H), 1.15-1.26 (m, 6H), 0.96 (s, 9H). DSC (50.0-300.0°; 05.00 °/min): integral: 1342.57 mJ; onset: 155.30°; peak: 169.25°, end set: 180.63°. TGA reveals no weight loss. X-ray powder diffraction pattern shows peaks at about 3.86, 7.62, 8.79, 9.66, 11.39, 11.44, 14.13, 14.19, 14.58, 15.18, 16.00, 16.59, 17.06, 17.55, 17.77, 18.09, 18.40, 19.00, 19.99, 20.69, 21.18, 22.37, 22.80, 23.72, 24.74, 25.08, 25.56, 26.67, 28.36, 28.55±0.2 °2θ.

### HPLC conditions set for analysis of samples:

HPLC analysis was performed using Shimazdu instrument, mobile phase used was Ammonium phosphate buffer+methane sulfonic acid [pH=4] and column used was Zorbax SB-CN, 4.6×250 mm. The detector wavelength was set to 210 nm. Throughout the analysis the flow rate was set to 1.5 ml per minute. Each sample (concentration 2000 ppm) was run for 60 min and the column oven temperature was set to 40° during analysis.

### Other analytical methods and description of instruments:

NMR spectra were obtained on a 400 MHz Bruker instrument, with CDCl_3_/DMSO-d_6_ as solvent. Chemical shifts (δ) are given in ppm relative to tetramethylsilane (δ = 0 ppm) or to residual protons in the solvent as internal standard. IR spectra were recorded with a PerkinElmer Spectrometer (Spectrum 100), and absorption bands are given in cm^−1^. TGA were recorded on PerkinElmer model Pyris 1 at the heating rate of 10 °/min and weight loss is given in percentage. DSC was recorded on Perkin Elmer model Diamond DSC at the heating rate of 10 °/min and endothermic peak is reported in ° and ΔH is reported in J/g. Melting point was recorded on Mettler Toledo apparatus with 1 °/min heating rate. Moisture content was determined by Karl Fischer titration method with a Metrohm instrument. Measurement of specific optical rotation was done on a Jasco, Model No. P1030 at the wavelength of 589 nm.

### Thermal stability studies:

Quinapril hydrochloride solvates (10 g each) of nitromethane, acetone, toluene, acetonitrile, methyl formate, dry quinapril hydrochloride, quinapril tris, quinapril hydrochloride, quinapril erbumine, quinapril magnesium salt(s)were kept at 60° and 80° in different drying ovens for 48 h. Samples of each were withdrawn after every 4 h for analysis of DKP impurity. Rates of degradation of quinapril to DKP were calculated by regression analysis, wherein coefficient of correlation was more than 98%. The ratio of rate of degradation of individual salt with that of dry quinapril hydrochloride was calculated (Table 2).

**TABLE 2:** RELATIVE THERMAL DEGRADATION RATES OF VARIOUS QUINAPRIL FORMS

Quinapril form	R at 60°	R at 80°
QHCl	1	1
QHCl toluene solvate	0.85	0.50
QHCl acetone solvate	0.36	0.38
QHCl acetonitrile solvate	0.34	0.28
QHCl methyl formate solvate	0.32	0.27
QHCl nitromethane solvate	0.20	0.16
Quinapril tris	0.008	0.003
Quinapril magnesium salt	0.85	0.59
Quinapril erbumine salt	1.28	1.73

R = Ratio of rate of formation of diketopiperazine in different forms of quinapril with respect to rate of formation of diketopiperazine in quinapril hydrochloride (QHCl).

### Solution Phase stability study:

For solution phase stability study quinapril hydrochloride/quinapril tris salt(s) (100 mg each) were dissolved in 1 ml each of water, 0.1N hydrochloride solution, 4.5 phosphate buffer solution, 6.8 phosphate buffer solution, 7.5 phosphate buffer solution in separate sample vials and kept for 30 min at 25°. Then each sample was analyzed by HPLC to detect impurity level. The results are provided in Table 3.

**TABLE 3:** COMPARATIVE SOLUTION PHASE STABILITY OF QUINAPRIL HYDROCHLORIDE AND TRIS AT DIFFERENT PH

Medium	Quinapril hydrochloride*		Quinapril tris*	
		
	DKP (%)	Diacid (%)	DKP (%)	Diacid (%)
Initial	0.20	0.08	0.04	0.02
D M Water	0.67	0.07	0.04	0.05
pH 0.1	0.44	0.28	0.08	0.03
pH 4.5	0.26	0.37	0.17	0.04
pH 6.8	0.47	0.37	0.05	0.05
pH 7.5	0.28	0.50	0.06	0.04

* amount of impurity (DKP and Diacid) formed (detected by HPLC) when the respective compound is kept for 30 min at 25° at different pH. DKP is diketopiperazine and Diacid, please refer to fig. 1.

### Single crystal X Ray Diffraction Analysis:

Single crystal of nitromethane solvate of quinapril hydrochloride was obtained by dissolving quinapril hydrochloride in nitromethane and allowing it to crystallize. Suitable crystal of quinapril tris was selected from the bulk for X-ray diffraction analysis. The crystallographic data and details of data collection of the compounds, *viz.* quinapril hydrochloride nitromethane solvate and quinapril tris, are given in Tables 4 and 5, respectively. Crystal of suitable size was selected and immersed in partone oil, then mounted on the tip of a glass fiber and cemented using epoxy resin. Intensity data for the compound was collected using MoK_α_ (λ = 0.71073Å) radiation on a Bruker Smart Apex diffractometer equipped with CCD area detector at 100K. The data integration and reduction was processed with SAINT[32] software. An empirical absorption correction was applied to the collected reflections using SADABS[33] program. The structure was solved by direct methods using SHELXTL[34] and was refined on *F^2^* by the full-matrix least-squares technique using the SHELXL-97[35] program package. All non-hydrogen atoms except ethyl carbon atoms of the ethyl acetate moiety (which are disordered) were refined anisotropically till convergence was reached. The occupancy factor for the disordered ethyl group was calculated by the FVAR command of the SHELXTL program and the disordered atoms were refined only isotropically. The hydrogen atoms of these disordered atoms were not included in the final refinement cycles. Hydrogen atoms attached to the ligand moieties were stereochemically fixed. The diagrams of the crystal structures were generated using the programs ORTEP5[36], Mercury 1.4.1[37] and PALTON[38].

**TABLE 4:** SUMMARY OF CRYSTALLOGRAPHIC DATA FOR NITROMETHANE SOLVATE OF QUINAPRIL HYDROCHLORIDE

Parameter	Value/Result/Description
Chemical formula	C_26_H_29_N_3_O_7_Cl_1_
Formula weight	530.97
Crystal Colour	Colourless
Crystal Size (mm^3^)	0.33 × 0.27 × 0.13
Temperature (K)	100
Crystal System	Orthorhombic
Space Group	P2_1_2_1_2_1_
a(Å)	10.462(2)
b(Å)	10.748(2)
c(Å)	24.186(5)
α(°)	90.0
β(°)	90.0
γ(°)	90.0
Z	4
V(Å^3^)	2719.4(9)
Density (Mg/m^3^)	1.297
Absorption Coefficient (mm^−1^)	0.188
F(000)	1116
Reflections Collected	14810
Independent Reflections	5327 [R(int) = 0.0866]
Number of Parameters	335
S (Goodness of Fit) on F^2^	1.049
Final R1, wR2 (I>2σ(I)	0.0904/ 0.1651
Weighted R1, wR2(all data)	0.1316/ 0.1840

**TABLE 5:** CRYSTAL DATA AND STRUCTURE REFINEMENT PARAMETERS OF QUINAPRIL TRIS

Parameter	Value/Result/Description
Chemical formula	C_29_H_41_N_3_O_8_
Formula weight	559.65
Temperature	100(2)K
Wavelength	0.71073 Å
Crystal system	Monoclinic
Space group	P2_1_
Unit cell Dimensions	a=10.401(3) Å, b=6.1709(18) Å, c=23.153(7) Å, ß=91.748(5)°
Volume	1485.4(7) Å^3^
Z	2
Density (calculated)	1.251 Mg/m^3^
Crystal size	0.45 × 0.40 × 0.10 mm^3^
Absorption coefficient	0.091 mm^−1^
F(000)	600
range for data collection	1.76 to 24.99°
Reflections collected	2863
Independent reflections	2091
Refinement method	Full-matrix Least-squares on F2
Data/restraints/parameters	2863/3/368
Goodness-of-fit on F2	1.008
Final R indices	R1=0.0619 [4431 *I>2(I)]*, wR2=0.1297
R indices (all data)	R1=0.0881, wR2=0.1396
Largest diff. peak and hole	0.320 and -0.234 e.Å^−3^

## RESULTS AND DISCUSSION

**Fig. 3:**
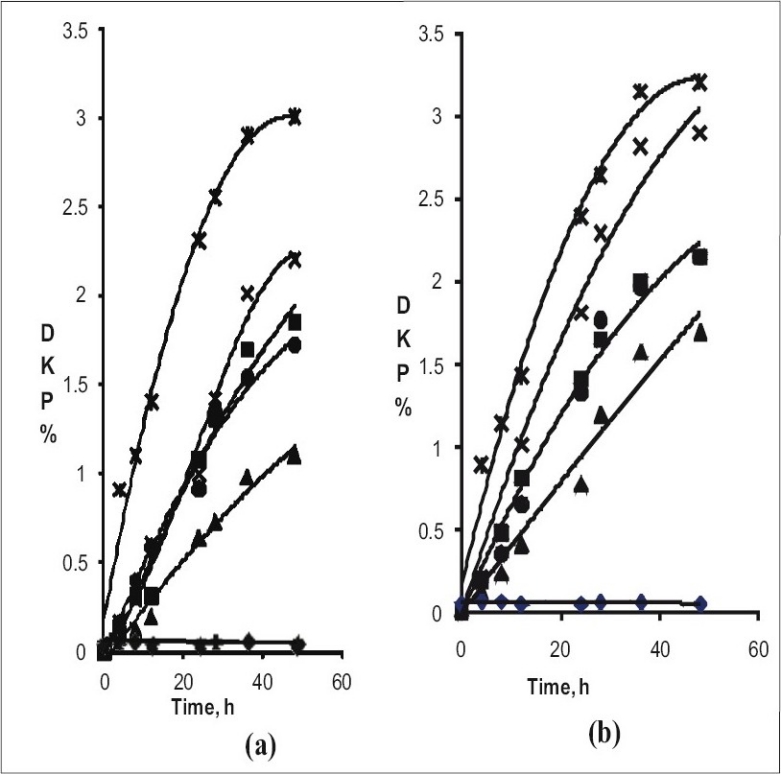
Solid state degradation study of quinapril solvates/salts. ■ Acetonitril, ▲ Nitromethane, × Acetone, ✳ Toluene, ● Methyl formate, ♦ Quinapril tris. (a) Degradation pattern of Quinapril solvates/ salts at 60° for 48 H, (b) Degradation pattern of Quinapril solvates/ salts at 80° for 48 H

Fig. 3 provides plot of percentage DKP impurity versus time when quinapril hydrochloride solvates and quinapril tris salt were kept at 60° and 80°, respectively. Rate of cyclization of quinapril to its DKP impurity is lowest in case of nitromethane solvate and highest in case of toluene solvate. Varying degradation rates observed in the solvates of quinapril hydrochloride reveal that the solvents embedded in the voids of quinapril hydrochloride crystal control the rate of escape of hydrogen chloride, the rate determining step in cyclization (fig. 2).

In spite of similarity in dipole moment between nitromethane (3.46 D) and acetonitrile (3.47 D), the nitromethane solvate is approximately twice as stable as that of acetonitrile solvate at 60°. Similarly, even though boiling points of nitromethane (101.2°) and toluene (110°) are closer, nitromethane solvate was found to be three times more stable than that of toluene solvate at 60°. To understand this phenomenon the powder and single crystal X-ray diffraction of the solvates of quinapril hydrochloride were studied. Powder X-ray diffraction pattern of all the solvates were found to be almost similar to each other, hence it is likely that the crystal systems of all these solvates would be similar.

**Fig. 4:**
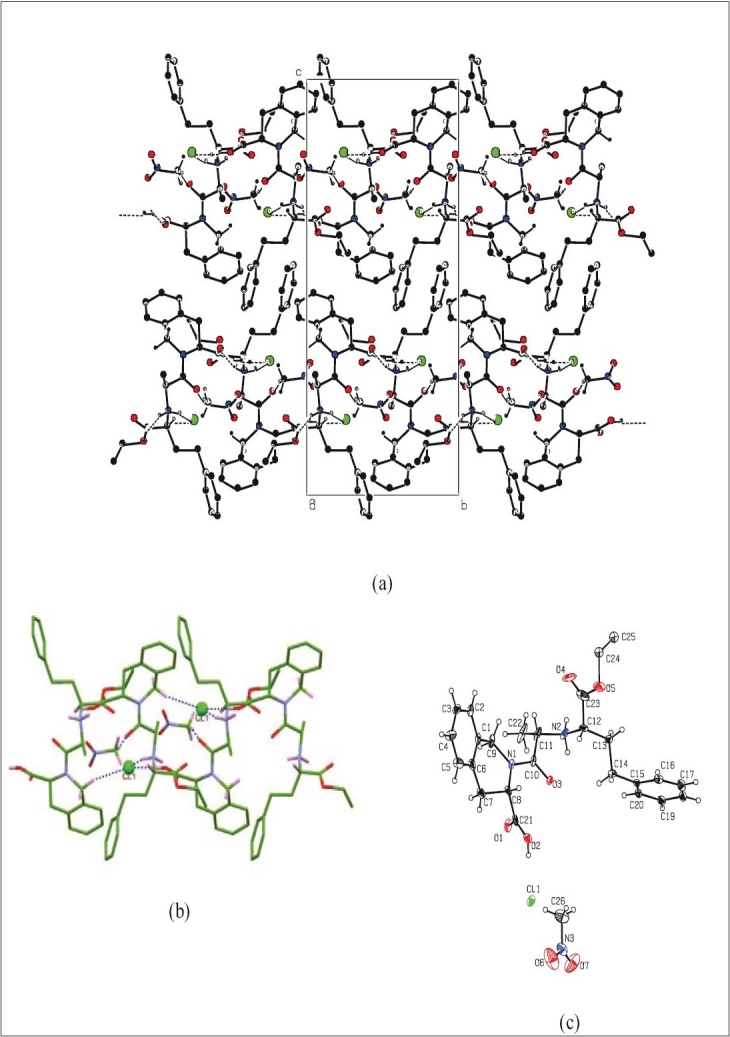
Single Crystal X-ray Diffraction study of quinapril hydrochloride nitromethane solvate. (a). Packing diagram with hydrogen bonding interaction viewed down a-axis depicting the H-bonded layered network for the compound. (b) Close-up view depicting the hydrogen bonding interaction of chloride anion and lattice nitromethane molecule in the 2_1_ screw related cleft (dotted blue line) by the quinapril monoanion by dimeric association. (c) ORTEP diagram with atom numbering scheme of the Quinapril hydrochloride along with lattice nitromethane molecule (30% probability factor for the thermal ellipsoids and only one position of the isotropic ethyl group of the ethyl acetate moiety is shown in the figure for clarity.)

ORTEP diagram of the quinapril nitromethane solvate is depicted in fig. 4c. Details of crystallographic data are summarized in Table 4. In an attempt to understand the interactions of the chloride and lattice nitromethane molecule with the quinapril monoanion, we have analyzed the packing and hydrogen bonding interaction of the compound in detail. Packing diagram of the compound with various hydrogen bonding interactions viewed down the a-axis for the compound is shown in fig 4a. Pairs of the protonated quinapril molecules are oriented with the flexible tethered phenyl terminal in opposite direction along c-axis to make effective intermolecular N-H…O interaction from either end between the protonated amine and the carboxylic acid oxygen O1 [N(2)…O(1)= 2.820(7) Å, < N(2)-H(2A)…O(1)= 164°] generating a cleft down a-axis. These dimeric protonated quinapril molecules are set along b-axis generating a layered network with 2_1_ screw related cavities arranged alternately. It is interesting to note that the chloride anion and the lattice nitromethane molecules via various hydrogen bonding interactions occupy these cavities. Close-up view depicting the encapsulation of the chloride ion and nitromethane molecule is shown in fig. 4c. Chloride anion is anchored inside the cavity by involvement of three hydrogen bonding interactions O-H…Cl between the carboxylic hydrogen H2, N-H…Cl with the protonated amine and C-H…Cl of the methylene hydrogen H2B of the six membered rings. The methyl hydrogen of the nitromethane present inside the cavity are involved in strong intermolecular C-H…O interaction (C(26)….O(3)= 3.514(8) Å < C(26)-H(26B)….O(3)= 171°) with the ketonic oxygen O3. In addition to the above interactions, weak intramolecular C-H…O contact exist between (i) ketonic oxygen O3 and the H12 attached to the asymmetric carbon and (ii) the acetate oxygen O4 with the H11 of the heterocyclic ring. Details of all these pertinent hydrogen-bonding interactions, along with symmetry code, are given in Table 6.

**TABLE 6:** HYDROGEN BONDING INTERACTIONS IN QUINAPRIL NITROMETHANE SOLVATE

D-H…A	(H…A)(Å)	(D…A) (Å)	<D-H..A (˚)
O(2)-H(2)…..Cl(1)^1^	2.19	3.013(5)	177
N(2)-H(2A)….O(1)^2^	1.94	2.820(7)	164
N(2)-H(2B)….Cl(1)^2^	2.43	3.180(5)	141
C(9)-H(9B)….Cl(1)^3^	2.77	3.713(6)	164
C(11)-H(11)….O(4)^1^	2.54	3.036(9)	111
C(12)-H(12)….O(3)^1^	2.49	2.972(6)	110
C(26)-H(26B)….O(3)^4^	2.56	3.514(8)	171

Symmetry code: 1. x, y, z; 2. 1-x, 1/2+y, 3/2-z; 3. x,1+y,z; 4. 2-x,-1/2+y,3/2-z; D: Hydrogen bond donor; A: Hydrogen bond acceptor; <: Bond angle

Hence, it is evident from the crystal structure analysis the hydrogen bonding interactions between nitromethane and quinapril hydrochloride molecule provide comparatively more stability to the product. However, such interactions are weak, if present, in case of acetonitrile solvate[39,40], and probably may not be present in the other known quinapril hydrochloride solvates as evident from their stability data.

Thermo gravimetric analysis of quinapril tris salt shows no weight loss. Differential scanning calorimetric analysis shows single endothermic peak at 156°, unlike quinapril hydrochloride showing first peak at 166° of degradation and a second peak corresponding to the melting point of the diacid impurity (III) of quinapril[8].

In FTIR spectrum (Table 1), the –OH stretching vibration (3709 cm^−1^) of carboxylic function in quinapril is seen in quinapril erbumine salt but absent in quinapril tris salt. Further, it can also be noted that the carbonyl stretching vibrations seen at the wavelengths of 1647 and 1648 cm^−1^ in quinapril erbumine salt and quinapril hydrochloride salt respectively are shifted to 1625 cm^−1^ in case of quinapril tris salt. Shifting of carbonyl stretch and absence of –OH stretch of carbonyl function of quinapril in quinapril tris salt is attributed to involvement of these groups in intermolecular hydrogen bonding.

**Fig. 5:**
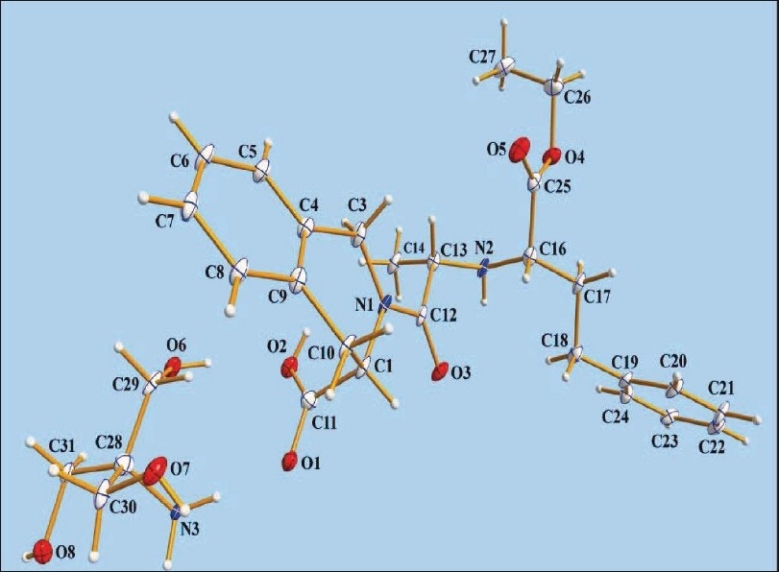
ORTEP view of quinapril tris with atom numbering scheme.

**Fig. 6:**
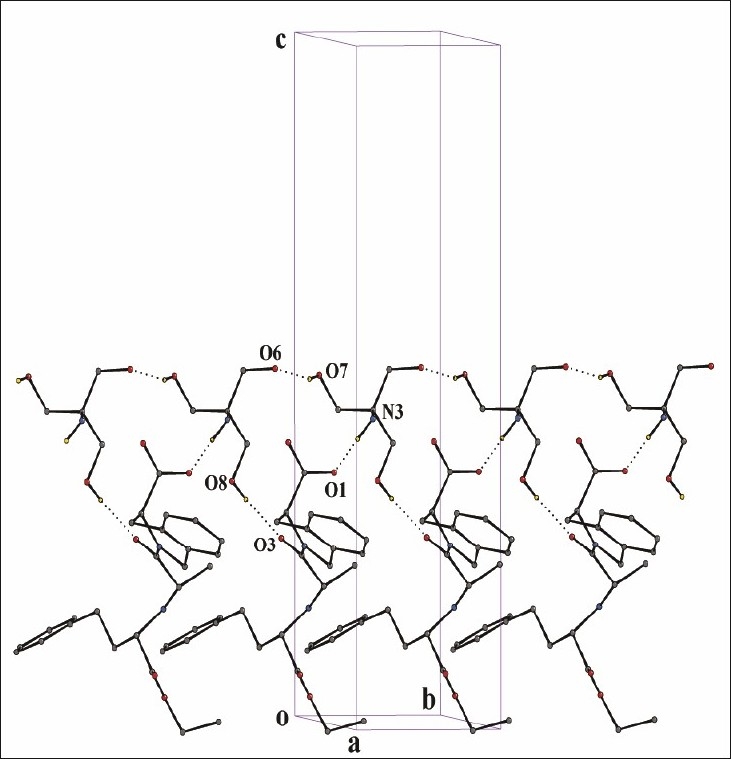
Quinapril tris crystal structure: One-dimensional chain propagating along the [010] direction.

**Fig. 7:**
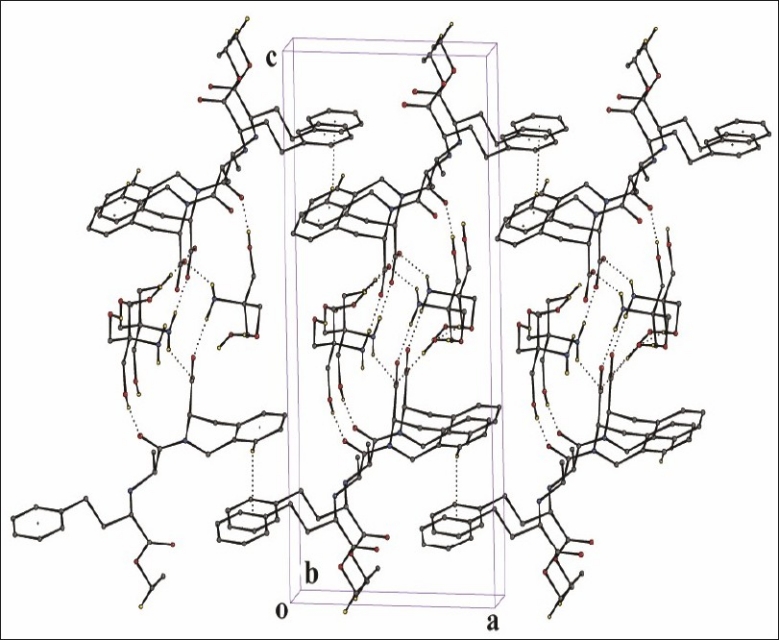
Quinapril tris crystal structure: Formation of three-dimensional supramolecular framework.

An ORTEP[37] view of asymmetric unit of quinapril tris salt with atom numbering scheme is shown in fig. 5. The conformation of the quinapril molecule is established by the torsion angles N1-C12-C13-N2 150.3(5)˚, N2-C16-C17-C18 -62.5(6)˚ and C16-C17-C18-C19 -167.8(5)˚. The hetero ring (C1/N1/C3/C4/C9/C10) assumes a screw-boat conformation with ring puckering parameters[41] Q=0.551(5) Å, θ=69.7(6)˚ and φ=-27.9(6)˚, respectively. The molecular structure of the title compound is stabilized by several intermolecular hydrogen bonds (Table 7) between quinapril and tris molecules. Intermolecular N(3)-H(31A)O(2), O(8)-H(8A)O(3) and O(7)-H(7A)O(6) hydrogen bonds connect the molecules into R33(18) rings which are corner fused to form one-dimensional polymeric chains propagating along the [010] direction (fig. 6). Adjacent one-dimensional chains are joined by O(6)-H(6A)O(2) and N(3)-H(31B) O(1) hydrogen bonds forming molecular channels which are further linked via C-H hydrogen bonds and interactions, so generating a three-dimensional framework (fig. 7).

**TABLE 7:** SELECTED INTERMOLECULAR CONTACTS IN QUINAPRIL TRIS

D-H…A	(H…A)(Å)	(D…A) (Å)	<D-H..A (°)
O(6)-H(6A)O(2)^i^	1.985(4)	2.785(5)	164.9(3)
N(3)-H(31B)O(1)^i^	1.793(4)	2.752(5)	175.4(2)
N(3)-H(31A)O(2)^ii^	2.137(4)	2.976(5)	145.1(2)
O(8)-H(8A)O(3)^iii^	1.968(4)	2.783(5)	172.7(2)
O(7)-H(7A)O(6)^iv^	2.053(4)	2.825(5)	156.9(3)
C(5)-H(5)Cg(1)^v^	2.86	3.583(7)	135
C(26)-H(26A)Cg(1)^vi^	2.83	3.564(8)	133
Cg(1)-Cg(2)^vii^		4.805(4)	
Cg(2)-Cg(1)^v^		5.249(4)	

Symmetry code: (i) x, y, z; (ii) -x+1, y-½, -z+1; (iii) -x+1, y+1/2, -z+1; (iv) x, y-1, z; (v) 1+x, 1+y, z; (vi) -x, ½+y, -z; (vii) -1+x, -1+y, z. Cg(1) and Cg(2) are the centroids of rings C19-C24 and C4-C9. D: Hydrogen bond donor; A: Hydrogen bond acceptor; <: Bond angle

It is evident from the single crystal X-ray diffraction and FTIR data, the interactions between quinapril and tris(hydroxymethyl)amino methane occurs by two phenomena *viz.* (a) formation of amine salt (b) complex intermolecular hydrogen bonding network. Hence, it imparts stability not only by the ionic bond but also through hydrogen bonds similar to co-crystal formation, which was not possible in quinapril erbumine and quinapril hydrochloride salts as evident from comparison of thermal stabilities of the quinapril tris salt and quinapril erbumine salt (Table 2)

Impurity levels in quinapril tris surprisingly remains unchanged at 80° even up to 72 h, however impurity levels in quinapril hydrochloride, quinapril erbumine and quinapril magnesium increased significantly. Rate of formation of DKP impurity in quinapril tris salt is the lowest (Table 2).

Solution phase stability study of these salts reveals significant increase in concentration of DKP and diacid impurity at different pH in case of quinapril hydrochloride after 30 min at 25°, however there is no significant change in the impurity level in case of quinapril tris salt (Table 3). Solubility study of quinapril tris salt and quinapril hydrochloride in water, ethanol and methanol at 20° reveals that quinapril tris salt has comparable solubility as that of quinapril hydrochloride (Table 8).

**TABLE 8:** COMPARISON OF SOLUBILITY OF QUINAPRIL TRIS SALT WITH HYDROCHLORIDE SALT

Solvent	Quantity of solvent required to dissolve 100 mg of	
	
	Quinapril hydrochloride*	Quinapril Tris*
Water	0.13 ml	0.1 ml
Methanol	1.4 ml	1.3 ml
Ethanol	2.2 ml	2 ml

* Volume of solvent (ml) required to dissolve 100 mg of respective compound at 20°.

The single crystal X-ray diffraction analysis of acetonitrile solvate of quinapril hydrochloride[39,40] does not show hydrogen-bonding interaction between the solvent molecule and the host. The solvent molecule just fit into the cavities of the crystal lattice to impart stability. On the other hand, nitromethane solvate of quinapril hydrochloride is comparatively more stable among all the other solvates due to strong hydrogen bonding interaction between nitromethane molecule and quinapril molecule, thereby imparting superior stability.

In quinapril tris salt, quinapril is not only bound with tris(hydroxymethyl)amino methane by the way of ionic bond, but also bound with a complex hydrogen bonding network involving large number of hydrogen bond donors and acceptors. This formation of complex hydrogen bonding network imparts solid-state stability as evident from comparison of thermal stabilities quinapril tris salt and quinapril erbumine salt, as *tert-*butylamine does not contain large number of hydrogen bond donors like that of tris (hydroxymethyl)amino methane. The interactions between quinapril and tris(hydroxymethyl)amino methane occurs by two phenomena *viz.* (a) formation of amine salt (b) complex intermolecular hydrogen bonding network. Hence, it imparts stability not only by the ionic bond but also through hydrogen bonds similar to co-crystal formation, which was not possible in quinapril erbumine and quinapril hydrochloride salts.

Quinapril tris salt would be a potential alternative to be formulated as a drug product to the existing solvates and salt forms of quinapril in view of its stability and comparable solubility to that of quinapril hydrochloride.

## References

[1] Steinberg SM, Bada JL (1983). Peptide decomposition in the neutral Ph region via the formation of diketopiperazines. J Org Chem.

[2] Chimanlal G, Ronals TB (1998). Kinetics of diketopiperazine formation using model peptides. J Pharm Sci.

[3] Purdie JE, Benoiton NL (1973). Piperazinedione formation from esters of dipeptides containing glycine, alanine and sarcosine: The kinetics in aqueous solution. Chem Soc Perkin Trans 2.

[4] Steinberg SM, Bada JL (1981). Diketopiperazine formation during investigations of aminoacid racemization in dipeptides. Science.

[5] Hoefle ML, Klutchko S (1982). Substituted acyl derivatives of 1,2,3,4-tetrahydroisoquinoline-3-carboxylic acids. US Patent No. 4,344,949.

[6] Jiyon TS, Jiefurii EB, Jiyon AR (1982). N-(substituted aminoalkanoyl)heterocyclic compounds. GB Patent No. 2,095,252.

[7] Patchett AA, Wu MT (1982). Isoquinoline carboxylic acid derivates, process for preparing and pharmaceutical composition containing the same. EP Patent No. 0,065,301.

[8] Guo Y, Byrn SR, Zografi G (2000). Physical characteristics and chemical degradation of amorphous quinapril hydrochloride. J Pharm Sci.

[9] Ulrich JG, Hilfiker R (2006). The importance of solvates. Polymorphism in pharmaceutical Industry.

[10] Brittain HG (2007). Polymorphism and solvatomorphism 2006. J Pharm Sci.

[11] Baure J, Morley J, Spanton S, Leusen FJ, Henry R, Hollis S (2006). Identification, preparation, and characterization of several polymorphs and solvates of terazosin hydrochloride. J Pharm Sci.

[12] Rollinger JM, Burger A (2002). Physicochemical characterization of hydrated and anhydrous crystal forms of amlodipine besylate. J Thermal Anal Calorimetry.

[13] Petit S, Coquerel G (1996). Mechanism of several solid–solid transformations between dihydrated and anhydrous copper(II) 8-hydroxyquinolinates. proposition for a unified model for the dehydration of molecular crystals. Chem Materials.

[14] Li J, Guo Y, Zugrufi G (2002). Effects of a citrate buffer system on the solid-state chemical stability of lyophilized quinapril preparations. Pharm Res.

[15] Harris M, Hokanson G, Murthy K, Reisch R, Waldman F (1988). Stabilized compositions. US Patent No. 4,743,450.

[16] Sherman BC (2003). US Patent No. Pharmaceutical compositions comprising quinapril magnesium.

[17] Klokkers K, Kramer KT, Fischer W, Sendl-Lang A (2004). Matrix controlled transdermal system for stabile derivatives of ace inhibitors. US Patent Application No. 20040052835.

[18] Klokkers K, Helfrich M, Nink J (2007). Matrix controlled transdermal system for stabile derivatives of ace inhibitors. WO, 2007065638.

[19] Li J, Guo Y, Zografi G (2002). The solid-state stability of amorphous quinapril in the presence of beta cyclodextrins. J Pharm Sci.

[20] Goel OP, Krolls U (1988). Crystalline quinapril and a process for producing the same. US Patent No. 4,761,479.

[21] Singh GP, Rawat GS, Dhake VN, Nehate SP (2005). Crystalline form of quinapril hydrochloride and process for preparing the same. EP1572661.

[22] Vishweshwar P, McMahon JA, Bis JA, Zaworotko MJ (2006). Pharmaceutical Co-crystals. J Pharm Sci.

[23] Etter MC, Adsmond DA (1990). The use of co-crystallization as a method of studying hydrogen bond preferences of 2-aminopyridine. J Chem Soc Chem Commun.

[24] Etter MC (1990). Graph-set analysis of hydrogen-bond patterns in organic crystals. Acta Crystallogr B.

[25] Etter MC, Urbanczyk-Lipkowska Z, Zia-Ebrahima M, Panunto TW (1990). Hydrogen bond directed co crystallization and molecular recognition properties of diarylureas. J Am Chem Soc.

[26] Carl HG, Hans PH (2000). On the inclusion of solvent molecules in the crystal structures of organic compounds. Acta Crystallogr B.

[27] SenthilKumar VS, Nangia A (2002). Molecular complexes of some mono- and dicarboxylic acids with *trans*-l,4,-dithiane-l,4-dioxide. Crystal Growth Design.

[28] Desiraju GR (2003). Crystal and co-crystal. Crystal Engg Commun.

[29] Almarsson O, Bourghol HM, Peterson M, Zaworotko MJ, Moulton B, Rodriguez-Hornedo N (2007). Pharmaceutical co-crystal compositions of drugs such as carbamazepine, celecoxib, olanzapine, itraconazole, topiramate, modafinil, 5-fluorouracil, hydrochlorothiazide, acetaminophen, aspirin, flurbiprofen, phenytoin and ibuprofen. US Patent Appl. 20070059356.

[30] Jennings SM (2005). Preparation of quinapril hydrochloride. US patent No. 6,858,735.

[31] Monsalvatje LM, Bartra SM, Tomas NJ, Puig TS (2003). Process for obtaining quinapril hydrochloride and solvates useful for isolating and purifying quinapril hydrochloride. US patent No. 6,617,457.

[32] Sheldrick GM (1995).

[33] (1997).

[34] Sheldrick GM (1997). SHELXTL reference manual: V*ersion 5.1*, Madison, Bruker AXS, WI.

[35] Sheldrick GM (1997).

[36] Spek AL (2003). PLATON for MS-Windows. Journal of Applied Crystallography.

[37] Farrugia LJ (1997). Ortep-3 for Windows. J Applied Crystallogr.

[38] Macrae CF, Edgington PR, McCabe P, Pidcock E, Shields GP, Taylor R (2006). Mercury: visualization and analysis of crystal structures. J Applied Crystallogr.

[39] Hausin RJ, Codding PW (1991). Molecular and crystal structure of MDL27,467A Hydrochloride and Quinapril Hydrochloride, Two Ester Derivatives of Potent Angiotensin Converting Enzyme Inhibitors. J Med Chem.

[40] The Cambridge Structural Database (CSD)- The world repository of small molecule crystal structures. The Cambridge Crystallographic Data Centre, Copyright © 2004-2008.

[41] Boeyens JCA (1978). Calculation of ring puckering parameters. J crystallogr Mol Structure.

